# Enrichment of Wheat–Chia Bread with Hemp, and Buckwheat Flours and *Cistus incanus* L. Infusion: Impact on Chemical Composition, Polyphenols, Fatty Acids, Amino Acids, and Consumer Acceptance

**DOI:** 10.3390/molecules31071198

**Published:** 2026-04-03

**Authors:** Anna Mikulec, Barbara Mickowska, Joanna Oracz, Kaja Karwowska, Magdalena Skotnicka, Stanisław Kowalski

**Affiliations:** 1 Faculty of Engineering Sciences, University of Applied Sciences in Nowy Sacz, 33-300 Nowy Sacz, Poland; 2 Department of Plant Product Technology and Nutrition Hygiene, Faculty of Food Technology, University of Agriculture in Krakow, 30-149 Krakow, Poland; barbara.mickowska@urk.edu.pl; 3 Institute of Food Technology and Analysis, Faculty of Biotechnology and Food Sciences, Lodz University of Technology, 90-924 Lodz, Poland; joanna.oracz@p.lodz.pl; 4 Department of Commodity Science, Faculty of Health Sciences, Medical University of Gdansk, 80-210 Gdansk, Poland; kajakarwowska@gumed.edu.pl (K.K.); skotnicka@gumed.edu.pl (M.S.); 5 Department of Carbohydrate Technology and Cereal Processing, Faculty of Food Technology, University of Agriculture in Krakow, 122 Balicka Street, 30-149 Krakow, Poland; st.kowalski@urk.edu.pl

**Keywords:** bread, *Cistus incanus* L., hemp flour, buckwheat flour, chia flour, polyphenols, fatty acids, amino acids, consumer acceptance

## Abstract

This study aimed to assess whether hemp or buckwheat flour, and the replacement of water with cistus infusion, can simultaneously improve the nutritional value and antioxidant potential of wheat–chia bread while maintaining acceptable sensory quality. Control bread (WCh) and variants with hemp flour (WChH) or buckwheat flour (WChB), prepared with either water or cistus infusion (Cis), were baked. The chemical composition, amino acid profile and protein quality (AAS), fatty acid profile, phenolic compounds and antioxidant properties (TPC, FRAP), color (CIELAB), and texture were determined. E-tongue and e-nose analyses, as well as consumer evaluation, were also performed. Hemp flour most significantly increased the protein and dietary fiber content of bread and improved the PUFA content and PUFA/SFA ratio. Buckwheat flour shifted the lipid profile toward MUFA and yielded the highest lysine AAS, although lysine remained the limiting amino acid in all variants. Cistus infusion increased the polyphenol pool and antioxidant activity, with the strongest effect observed in the combined WChH/Cis and WChB/Cis systems. Electronic nose and an electronic tongue analyses confirmed significant differentiation of the flavor and aroma profiles among variants. Consumer evaluation showed a decrease in acceptance following hemp flour addition, which was partially mitigated by cistus infusion, while buckwheat variants maintained good sensory acceptance.

## 1. Introduction

Among wheat cereal products, bread remains the most widely consumed [1]. It is widely recognized as one of the key staple foods globally [2], constituting an important part of the daily diet in many countries [3]. It is readily chosen by consumers regardless of their place of residence and socio-economic status, due to its convenience, favorable sensory properties, appropriate quality, relatively low price, and the lack of significant socio-cultural and religious restrictions related to its consumption [4]. However, traditional bread made from refined wheat flour is characterized by relatively low nutritional value due to its limited content of dietary fiber, minerals, and polyphenols, and its proteins are deficient in essential amino acids [3,5,6,7,8]. Hence, wheat bread is often defined as a food product with an unbalanced nutritional profile [9]. Such unbalanced diets have been shown to potentially contribute to malnutrition worldwide [10]. In response to the growing interest in food fortification, numerous studies have been conducted on enriching bread with ingredients of high nutritional and health-promoting value [11,12,13,14,15,16]. Bread made from refined flour has repeatedly proven to be a good carrier of functional ingredients and represents one of the best matrices for food enrichment. To date, wheat bread has been enriched with, among others, legume seeds [17,18], fruits and vegetables [19], herbs [20,21], roots and tubers [22], cereals other than wheat [23,24], oilseeds [15,25] and insects [26,27,28]. The introduction of these components into the bread recipe aims to improve the chemical composition of the bread while maintaining an acceptable sensory quality, which is crucial from the consumer’s perspective.

For example, chia seeds (*Salvia hispanica* L.) are a valued functional ingredient due to their high content of n-3 fatty acids (α-linolenic acid), dietary fiber, and protein. Their addition to bread dough has a beneficial effect on the lipid profile of bread by increasing the proportion of polyunsaturated fatty acids and lowering the n-6/n-3 fatty acid ratio in the finished bread [25]. Hemp flour, derived from *Cannabis sativa* L. seeds, is a rich source of protein with a favorable amino acid composition, insoluble dietary fiber, key micronutrients, and a well-balanced supply of both n-6 and n-3 polyunsaturated fatty acids, which may contribute positively to the lipid quality of enriched bakery products [15,29]. Common buckwheat (*Fagopyrum esculentum*) flour represents another nutritionally valuable option for wheat bread fortification. Unlike wheat, buckwheat protein is considered nutritionally complete, with a particularly high lysine content, an amino acid that is characteristically deficient in wheat-based products and thus represents the first limiting factor in their protein quality [30]. Whole grain buckwheat flour additionally contributes meaningful quantities of dietary fiber, trace elements including Fe, Zn, and Se, and a range of bioactive polyphenolic compounds with documented antioxidant activity [30,31]. One nutritional consideration associated with buckwheat is the presence of phytic acid, an antinutrient capable of chelating divalent minerals and thereby reducing their bioavailability [23]. *Cistus incanus* L., known as rockrose, is a Mediterranean shrub whose leaves and herb have long been used in herbal infusions for their health-promoting properties [32]. Cistus infusion is characterized by an exceptionally high content of polyphenols (predominantly flavan-3-ols (catechins), flavonols, and hydroxycinnamic acid derivatives), thanks to which it has strong antioxidant, antibacterial, and anti-inflammatory effects [32]. Incorporating cistus extract or infusion into food is regarded as an innovative way to fortify products with antioxidants [32,33,34]. In the context of baking, partially replacing water with cistus infusion can significantly increase the polyphenol content of both dough and finished bread, thereby enhancing antioxidant capacity and extending the microbiological shelf life of the bread [34]. Previous studies have widely investigated the enrichment of bread with individual functional ingredients, including plant-based and functional additives [2,9,11,13,14,16,17,18,20,27,28,29,30,32]. In our previous research, we also focused on enriching bread with selected components, including chia seeds [25], hemp flour [15,35], buckwheat flour [23], and cistus infusion [34], analyzing their effect separately. To the best of our knowledge, however, there are no comprehensive studies evaluating the combined use of these unconventional ingredients in bread making. It may be assumed that the combined use of multiple bioactive raw materials may result in a synergistic effect in terms of nutritional and antioxidant value, but their combined impact on the nutritional value and consumer acceptance of bread remains unknown. Therefore, this study aimed to holistically enrich wheat–chia bread with multiple additives simultaneously and comprehensively evaluate the resulting products. Wheat–chia bread was used as the control formulation, as chia flour had been previously identified as a nutritionally valuable and technologically suitable component in earlier studies. Therefore, chia was treated as a constant element of the base matrix rather than an experimental variable.

The aim of this study was to determine the effect of additional enrichment with hemp flour, buckwheat flour, and *Cistus incanus* L. infusion within a wheat–chia bread system, with particular emphasis on the chemical composition and polyphenol, fatty acid, and amino acid profiles, as well as consumer sensory acceptance.

## 2. Results and Discussion

### 2.1. Chemical Composition

The raw materials differed significantly in their chemical composition (*p* < 0.05) (Table 1). Hemp flour had the highest protein and total dietary fiber content (33.75 and 41.10 g/100 g, respectively), exceeding both chia flour (28.28 and 37.14 g/100 g) and buckwheat flour (14.35 and 3.52 g/100 g). In the bread samples, the addition of hemp flour (WChH) significantly increased the protein content to 14.86 g/100 g and total dietary fiber to 9.67 g/100 g, compared to the control bread (WCh) (11.39 and 3.47 g/100 g, respectively), mainly due to an increase in its insoluble fiber fraction (7.72 vs. 2.01 g/100 g; *p* < 0.05) (Table 1). The observed increase in protein and dietary fiber content in bread enriched with hemp flour is directly related to the high nutritional value of this raw material, particularly its high protein and insoluble fiber content, which has been consistently reported in previous studies [15,35,36]. Similar effects have been observed by Mocanu et al. [29], who reported a significant increase in protein and fiber content in wheat-based bakery products following hemp flour incorporation, confirming its strong fortification potential. Rusu et al. [37] reported that the incorporation of hemp flour at both 10% and 20% substitution levels significantly increased the protein content of bread compared to the wheat control, regardless of the variety used. Moreover, Del Vecchio et al. [38] demonstrated that hemp flour enrichment leads to a substantial improvement in the nutritional profile of bread, including increased protein and bioactive compound content, which is consistent with the trends observed in the present study. The addition of buckwheat flour (WChB) resulted in a significantly smaller enrichment effect (total fiber 3.93 g/100 g, compared to the control 3.47 g/100 g; protein 11.90 g/100 g compared to the control 11.39 g/100 g; *p* < 0.05), which is consistent with the results of other authors [25,30]. Similar trends were reported by Nakov et al. [39], who observed only moderate increases in protein and dietary fiber content following the incorporation of whole buckwheat flour, indicating a less pronounced fortification effect compared to other plant-based ingredients. This relatively limited increase may be attributed to the compositional characteristics of buckwheat flour, which, despite its high nutritional quality, contains lower amounts of dietary fiber compared to hemp flour and contributes less substantially to the overall protein content of the final product.

### 2.2. Amino Acid Composition and Protein Nutritional Quality

The amino acid profile of the bread differed significantly depending on the types of additives used (*p* < 0.05) (Table 2). Compared to the control bread (WCh), breads with hemp flour (WChH) and buckwheat flour (WChB) were characterized by significantly higher lysine content (27.27–27.29 and 28.70–28.95 vs. 23.03–23.98 mg/g protein, respectively), while bread with hemp flour also had higher methionine content (17.09–16.98 vs. 15.42–14.80 mg/g protein, respectively). Breads with hemp and buckwheat flour were also characterized by significantly higher total content of essential amino acids (EAAs) (Table 2). The observed increase in lysine and total essential amino acid (EAA) content in bread enriched with chia, hemp, and buckwheat flour is consistent with the known amino acid profiles of these raw materials (Table S1), particularly their higher lysine content compared to wheat proteins [12,23,29,30,38]. This effect is especially pronounced in the case of hemp flour, which is characterized by a well-balanced amino acid composition and high protein content. These findings align with studies by House et al. [40] and Oseyko et al. [41], which demonstrated that hemp seed and its derived products provide a complete essential amino acid profile meeting human nutritional requirements. Del Vecchio et al. [38] reported that bread enriched with hemp flour at 10–15% substitution level showed a markedly improved essential amino acid profile, including lysine, leucine, and phenylalanine, compared to control wheat bread. Similar findings were reported in hemp-enriched bakery products, where a substantial increase in protein content and essential amino acid availability was observed, confirming the strong fortification potential of this raw material [37]. The incorporation of buckwheat flour into wheat-based systems has been shown to increase the content of several essential amino acids, including leucine, methionine, threonine, and valine, as demonstrated by Coțovanu et al. [30], who reported a considerable increase in total amino acid content in wheat–buckwheat bread compared to wheat bread. These findings are consistent with the present study, where the addition of buckwheat flour resulted in a moderate but significant improvement in selected amino acids. The contribution of chia flour to EAA content was less pronounced, which may be related to its lower protein content compared to hemp flour. Despite these improvements, lysine remained the limiting amino acid in all variants, which is characteristic of cereal-based products. This indicates that although enrichment improves protein quality, it does not fully overcome the intrinsic limitations of wheat proteins, as also reported in previous studies on composite bread formulations [15,23,30].

In the group of non-essential amino acids (non-EAAs), the addition of hemp and buckwheat flours caused a significant increase in arginine and aspartic acid, with a simultaneous decrease in glutamic acid (Table 2). These changes are consistent with the characteristic amino acid composition of hemp and buckwheat flours [23,30,35,38]. This observation is further supported by studies conducted on bread systems, confirming the applicability of the observed trends in real bakery products.

In all bread, lysine remained the limiting amino acid (the AAS of lysine was the lowest among all assessed EAAs) (Table 3), which is characteristic of wheat-based bread and cereal blends [23]. The type of flour used significantly influenced the lysine AAS. Compared to the WCh control (47.97%), significantly higher lysine AAS values were obtained for the following breads: WChH (56.81%), WChH/Cis (56.85%), WChB (59.80%), and WChB/Cis (60.31%) (Table 3). Overall, the results indicate that the additives partially improved the nutritional quality of protein primarily by increasing the lysine AAS, although lysine was not eliminated as the limiting amino acid in any variant.

### 2.3. Determination of Fatty Acid Profile

The fatty acid composition of the bread significantly depended on the type of additives used (*p* < 0.05), and the observed changes were closely related to the fatty acid profiles of the applied ingredients (Table 4 and Table S2).

The content of saturated fatty acids (SFAs) was significantly lower in the hemp-enriched breads (WChH: 14.10 and WChH/Cis: 14.41 g/100 g) compared to the control samples (WCh 18.15 and WCh/Cis 18.10 g/100 g) (Table 4). In contrast, the SFA content in the buckwheat variants remained at a similar level (WChB 18.24 and WChB/Cis 18.40 g/100 g). These differences reflect the lower SFA fraction in hemp flour relative to wheat and buckwheat flour.

The content of monounsaturated fatty acids (MUFAs) was highest in the buckwheat enriched breads (WChB 24.58 and WChB/Cis 24.67 g/100 g), compared to 15.2 and 14.78 g/100 g in WCh and WCh/Cis, respectively (Table 4). This increase was mainly associated with a higher proportion of C18:1 cis (22.84–22.91 g/100 g vs. 13.35 g/100 g in WCh), consistent with the lipid profile of buckwheat flour, in which oleic acid is the dominant MUFA component (Table S2). A similar MUFA increase in buckwheat enriched bread systems has been reported by Nakov et al. [39], who showed that increasing the proportion of whole-grain buckwheat flour (10–50%) resulted in a gradual increase in total MUFA content in bread lipids, with oleic acid as the dominant MUFA fraction.

In turn, the polyunsaturated fatty acid (PUFA) fraction was most strongly affected by the addition of hemp flour. The PUFA content increased from 66.64 (WCh) and 67.12 g/100 g (WCh/Cis) to 72.72 (WChH) and 72.16 g/100 g (WChH/Cis) (Table 4). This resulted in the highest PUFA/SFA ratios in these variants (5.16 and 5.01, respectively), compared to 3.67 and 3.71 in the control samples. The observed increase was primarily due to higher levels of C18:2 n-6 cis (51.66–51.70 g/100 g vs. 44.97 g/100 g in WCh) and the presence of C18:3 n-6 (2.01–2.03 g/100 g), reflecting the fatty acid composition of hemp flour (Table S2). These findings are consistent with the results reported by Rusu et al. [37], who demonstrated that the incorporation of hemp flour into wheat bread increases the content of unsaturated fatty acids, particularly linoleic (C18:2 n-6) and α-linolenic (C18:3 n-3) acids, which are the dominant components of hemp lipids. This is further supported by Montero et al. [42], who highlighted that these two fatty acids account for the majority of the lipid fraction in hemp seeds and are primarily responsible for the nutritional enhancement observed in hemp-enriched food products. In contrast, the PUFA content in the buckwheat variants was lower (57.07 in WChB and 56.93 g/100 g in WChB/Cis), with corresponding PUFA/SFA ratios of 3.13 and 3.09, respectively. This is consistent with the fatty acid profile of buckwheat, which is characterized by a relatively lower proportion of polyunsaturated fatty acids and a higher contribution of monounsaturated fatty acids [39].

Significant differences were also observed in the n-6/n-3 ratio (*p* < 0.05). The lowest values were recorded for the control samples (WCh 2.08; WCh/Cis 1.92), associated with relatively high n-3 content (21.64 and 23.02 g/100 g, respectively) and moderate n-6 levels (44.99 and 44.10 g/100 g). In contrast, the hemp variants exhibited higher n-6/n-3 ratios (2.84–2.92), resulting from increased n-6 content (53.75–53.76 g/100 g) and reduced n-3 levels (18.41–18.96 g/100 g). The buckwheat-enriched bread showed intermediate values (2.58–2.59), with the lowest n-3 content (15.88–15.94 g/100 g) and n-6 levels of approximately 41 g/100 g (Table 4). From a nutritional perspective, both the PUFA/SFA ratio and the n-6/n-3 ratio are considered key indicators of lipid quality in food products. In the present study, the high PUFA/SFA ratios observed in the hemp-enriched breads, together with n-6/n-3 values remaining within nutritionally acceptable ranges, indicate an overall improvement in lipid quality. A higher PUFA/SFA ratio is associated with beneficial effects on cardiovascular health, including the reduction of LDL cholesterol levels, whereas a balanced n-6/n-3 ratio plays a crucial role in regulating inflammatory and metabolic processes [43]. These findings are consistent with current nutritional recommendations emphasizing the importance of fatty acid balance in functional food design [43,44].

Overall, these results demonstrate that hemp flour promotes a PUFA-dominant lipid profile, whereas buckwheat flour induces a relative shift towards MUFA, reflecting the inherent differences in the fatty acid composition of these raw materials and their distinct roles in shaping the nutritional quality of enriched bread.

### 2.4. Antioxidant Properties and Polyphenol Profile

Compared to WCh, the use of cistus infusion significantly increased the total phenolic content (TPC) and the antioxidant activity (FRAP) of the bread (*p* < 0.05). In the WCh/Cis sample, TPC increased from 50.27 to 71.26 mg GAE/L, and FRAP from 7.82 to 12.66 mg Trolox/L. The strongest antioxidant response was observed in variants combining cistus infusion with hemp or buckwheat flour. WChH/Cis and WChB/Cis exhibited significantly higher TPC (108.61 and 83.24 mg GAE/L, respectively) and FRAP values (19.71 and 18.67 mg Trolox/L) compared to both the control and the corresponding variants without infusion (*p* < 0.05). These results indicate that cistus infusion plays a key role in enhancing the antioxidant potential of the bread, while the presence of hemp flour further strengthens this effect. In the case of hemp flour, the effect appears to be synergistic, as this raw material itself is characterized by high antioxidant potential (Table S4), but only its combination with cistus led to a simultaneous increase in both TPC and FRAP. The buckwheat variants showed a less pronounced response; however, the addition of cistus still resulted in a significant improvement in antioxidant activity, indicating an additive effect. The changes were consistent with the polyphenol profile of the raw material, which was characterized by a high content of phenolic compounds and strong reducing potential (Table S3). The direct transfer of compounds from the infusion to the bread matrix was confirmed by the presence of epigallocatechin gallate (2.01 mg/100 g), quercetin (2.00 mg/100 g), and kaempferol (6.71 mg/100 g), which were absent in the control samples. Simultaneously, a significant increase in selected phenolic acids was observed, particularly sinapic acid (2.94 vs. 1.49 mg/100 g in WCh), indicating an important contribution of hydroxycinnamic derivatives to the antioxidant response. Taken together, the data from Table 5, in conjunction with the raw material profile (Tables S3 and S4), indicate that cistus infusion is primarily responsible for the qualitative enrichment of the bread’s polyphenol profile, while hemp flour enhances its functional effectiveness in the product matrix, resulting in the most pronounced synergistic effect among the systems analyzed. Similar trends have been reported in the literature. Del Vecchio et al. [38] and Ertaş and Aslan [45] observed that the addition of hemp flour increases both total phenolic content and antioxidant activity in bakery products. Likewise, Mumtaz et al. [46] observed a significant increase in antioxidant activity with the addition of buckwheat components to bread, confirming the potential of these raw materials in improving the functional properties of cereal-based products.

From a nutritional and functional perspective, total phenolic content (TPC), antioxidant activity measured by FRAP, and the detailed polyphenol profile are widely recognized as key indicators of antioxidant potential in food products [47,48,49]. Phenolic compounds are considered the main contributors to the reducing capacity of cereal-based systems [47,50], while their qualitative composition, including the presence of flavonoids and phenolic acids, plays a crucial role in shaping the overall antioxidant response of food matrices.

### 2.5. Electronic Nose and Electronic Tongue Analysis

**Figure 1:**
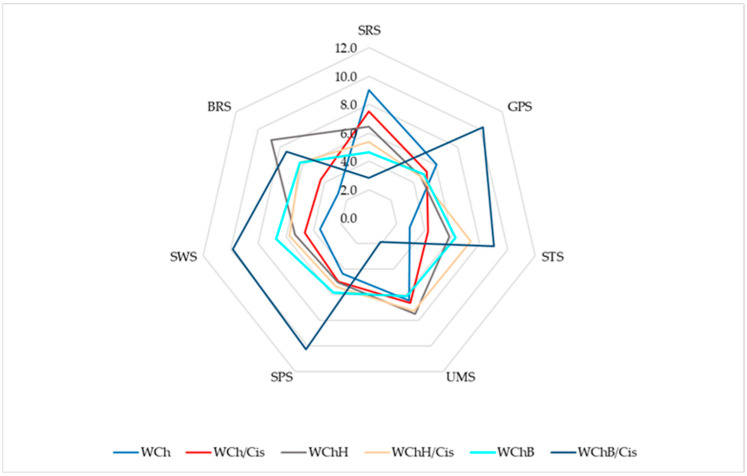
E-tongue sensor responses for extracts of the tested breads. Explanatory notes: SRS—sour taste, GPS—general-purpose sensor, STS—salty taste, UMS—umami taste, SPS—spicy taste, SWS—sweet taste, BRS—bitter taste; WCh—wheat–chia bread (control); WCh/Cis—wheat–chia bread with cistus infusion; WChH—wheat–chia bread with hemp flour; WChH/Cis—wheat–chia–hemp bread with cistus infusion; WChB—wheat–chia bread with buckwheat flour; WChB/Cis—wheat–chia–buckwheat bread with cistus infusion.

Instrumental sensory analysis revealed significant differences in the flavor and aroma profiles of the tested breads depending on the additives used (Figure 1). Electronic tongue analysis indicated that the standard (WCh) was characterized by the highest sour taste intensity (SRS) and low intensity of the other taste modalities. Replacing water with cistus infusion (WCh/Cis) reduced sourness and increased umami (UMS) and salty (STS) taste intensity. The addition of hemp flour (WChH) led to a further decrease in sourness and increased umami and bitter (BRS) taste intensity, while the WChH/Cis variant demonstrated a more balanced taste profile. Breads with buckwheat flour (WChB) were characterized by a higher intensity of sweet (SWS) and spicy (SPS) taste, while the combination of buckwheat and Cistus (WChB/Cis) resulted in the highest salty, spicy, and sweet flavor intensity with low sourness.

The volatile compound profile (Table 6) was dominated by ethanol (approx. 69–72% of the total volatile fraction), and its proportion, similarly to that of acetaldehyde, 1-propanol, and 3-methylbutanal, did not differ significantly between the variants, indicating the retention of a comparable “fermentative-alcoholic” aroma base. The addition of hemp and buckwheat flour increased the content of pyrazines (2,5-dimethylpyrazine and trimethylpyrazine) and 2-methylpropanal. Pyrazines are commonly reported as key products of Maillard-type reactions occurring during baking, resulting from interactions between amino acids and reducing sugars [51,52]. Importantly, the exact contribution of endogenous amino acids from the raw materials was not directly assessed, and thus the formation of these compounds should be interpreted as the result of combined effects of formulation and thermal processing rather than the direct transfer of specific constituents. The highest content of esters and aromatic aldehydes was found in WChB/Cis (2-phenylethyl acetate 1.62% and benzaldehyde 0.80%). An increase in the n-butanol content was also observed in WChH/Cis (Table 6).

Taken together, the e-tongue and e-nose results demonstrate that the applied additives influenced the overall flavor and aroma profile of the breads and allowed clear differentiation between the tested variants using instrumental sensory analysis. The observed differences in volatile composition should not be attributed solely to the raw materials, as many aroma-active compounds, particularly pyrazines and selected aldehydes, are formed during baking through Maillard and Strecker reactions. Therefore, the final sensory profile reflects a combined effect of ingredient composition, fermentation processes, and heat-induced transformations. In this context, the higher levels of selected volatiles in enriched variants may be associated with differences in precursor availability; however, this relationship was not directly assessed in the present study. The results are in agreement with previous findings on bread systems, where the incorporation of hemp flour was shown to modify the volatile compound profile and sensory characteristics of the final product [35,38].

### 2.6. Color Analysis

All recipe modifications caused significant color changes (*p* < 0.05) relative to the control (WCh) (L* 62.10; a* 1.22; b* 14.88) (Table 7). The strongest effect was observed in the variants with hemp flour: a significant decrease in lightness (L*) from 42.11 to 41.37 and the greatest total color differences (ΔE 20.51–21.23), indicating a clearly different product appearance. Replacing water with cistus infusion also significantly darkened the bread and shifted the color towards red and yellow (e.g., WCh/Cis: L* 55.75; a* 4.09; b* 20.89; ΔE 9.20). Buckwheat variants showed moderate changes, ranging from L* 56.92 and ΔE 5.35 (WChB) to L* 52.30 and ΔE 11.63 (WChB/Cis), with a simultaneous increase in a* and b* (e.g., WChB/Cis: a* 4.66; b* 20.11) (Table 7). These trends are consistent with previous studies on enriched cereal products, where the incorporation of plant-based ingredients rich in phenolic compounds and natural pigments leads to darker color and modified red–yellow tones [14,19,22]. Similar effects have also been reported for hemp- and cistus-enriched bread systems [15,34,35]. In addition, phenolic compounds may contribute to color development both directly, as natural pigments, and indirectly, through interactions with proteins and carbohydrates during thermal processing. Therefore, the final color of the enriched breads should be interpreted as the result of combined effects of raw material composition and heat-induced reactions, rather than a contribution solely of the added ingredients.

### 2.7. Texture Analysis

The addition of hemp and buckwheat flour significantly (*p* < 0.05) increased the hardness and chewiness of bread compared to the control (Table 8). The highest values were observed for WChB (Hardness 22.05 N; Chewiness 16.92) and WChH (19.85 N; 16.97), with a simultaneous decrease in cohesiveness in WChH (0.81 vs. 0.85) and in springiness (0.95 vs. 1.00). Replacing water with cistus infusion mitigated these effects and significantly reduced the hardness and chewiness of the enriched bread (e.g., WChB/Cis hardness 16.87 vs. 22.05 N in WChB; *p* < 0.05). The highest moisture content was determined in WChB/Cis (43.90%), and the lowest in WCh (41.42%), and these differences coincided with the direction of texture changes (Table 8). These findings are consistent with previous studies on composite bread systems, where partial replacement of wheat flour weakens the gluten network and results in a firmer crumb structure. Similar effects have been reported for hemp enriched products by Mocanu et al. [29] and Švec et al. [53], who observed increased hardness and reduced cohesiveness in enriched bakery products. Comparable trends have also been reported for buckwheat-enriched bread. Eren and Akkaya [54] demonstrated that the incorporation of buckwheat significantly increases bread hardness, while Coțovanu et al. [30] showed that buckwheat addition leads to structural changes in the dough and crumb associated with gluten dilution. Overall, the texture of the enriched breads reflects the combined effects of protein dilution, fiber content, and water availability within the crumb structure.

### 2.8. Consumer Acceptance

In the consumer acceptance assessment, the highest scores were obtained for the control variants (WCh and WCh/Cis), which were practically at the same level for all assessed attributes with no significant differences between these two samples (*p* < 0.05) (Table 9). The addition of hemp flour significantly decreased the scores for all parameters, most significantly for taste (WChH 2.80 points) and overall acceptance (WChH 3.25 points). Replacing water with cistus infusion in the hemp variant partially mitigated this effect, and scores increased to 3.35 points was observed for taste and 3.49 points for overall acceptance. Variants with buckwheat flour did not differ significantly from the control and maintained good acceptance (overall acceptance 4.46 points), with only a slight decrease relative to the control, mainly in taste (4.55 points) and crumb appearance and structure (4.26 points) (Table 9). The buckwheat bread with cistus infusion (WChB/Cis) differed significantly from the control in terms of taste (4.25 points) and overall acceptance (4.32 points). However, it’s of overall acceptance did not differ significantly from that of the hemp bread variants (Table 9). No significant differences were found for smell, appearance, and structure of the crust or crumb. The results for bread containing hemp flour confirm the so-called “sensory cost” associated with hemp flour inclusion in bread recipes, as reported in the literature. Similar results were obtained by other authors [35,38,53,55], who also observed lower scores for bread containing hemp flour and indicated a persistent bitter aftertaste as the main factor reducing the consumer attractiveness. The results for buckwheat bread are also consistent with those reported by Coţovanu et al. [30] and Eren and Akkaya [54], who obtained high sensory acceptance scores for breads containing buckwheat flour. In particular, Eren and Akkaya [54] indicated that the incorporation of 20% buckwheat flour into white bread formulations resulted in the most favorable balance between nutritional and sensory properties. Moreover, variants with an appropriately selected addition level and particle size of buckwheat flour were even preferred over the control.

The results of the consumer acceptance assessment clearly reflected the instrumentally recorded changes, both in color and texture, as well as in the e-nose and e-tongue profiles. These relationships should be considered primarily as a co-occurrence of characteristics, rather than evidence of a clear causal mechanism, but they do form a coherent picture of differences between the bread variants. Hemp bread were characterized by the strongest color shift compared to the control (WChH: L* 42.11; ΔE 20.51 and WChH/Cis: L* 41.37; ΔE 21.23 vs. WCh: L* 62.10) (Table 7). They were also characterized by significantly higher hardness and chewiness compared to the control bread (WChH: Hardness 19.85 N; Chewiness 16.97 N vs. 15.47 N and 13.06 N in WCh) (Table 8). At the same time, an increase in pyrazines was observed in the volatile profile (Table 6). Pyrazines are Maillard reaction products that influence the aroma, taste, and overall sensory quality of baked goods [56,57]. The increased pyrazine content in breads with hemp and buckwheat flour may be associated with more intensive Maillard reactions, likely related to differences in the availability of amino compounds compared to the control bread; however, this mechanism was not directly assessed in the present study [56,57]. The 2,5-dimethylpyrazine content increased from 0.26% in the control to 0.52–0.54% in the hemp and buckwheat variants, and trimethylpyrazine increased from 0.28% to 0.36–0.37% (WChH and WChH/Cis, respectively) and 0.38% (WChB/Cis). These compounds, depending on their structure and concentration, can enhance toasted, roasted, and earthy aroma notes [58]. At the same time, the hemp variants showed the greatest decrease in consumer scores, especially for taste and overall acceptance (WChH: taste 2.80, overall acceptance 3.25; WChH/Cis: taste 3.35, overall acceptance 3.49 vs. 4.75 and 4.74 in the control, respectively) (Table 9). This trend is consistent with the e-tongue results, which showed an increase in bitterness (BRS) and umami (UMS) intensity and a decrease in sourness for WChH compared to WCh (Figure 1). Replacing water with cistus infusion in the hemp variant partially mitigated the negative effect, as reflected in reduced texture hardness, improved crumb perception, and a more balanced e-tongue profile.

Breads with buckwheat flour maintained good overall acceptance (4.46–4.32) (Table 9), even though the WChB variant had the highest hardness (22.05 N) (Table 8), suggesting that hardness alone did not determine consumer acceptance. Additionally, the WChB/Cis variant exhibited greater aromatic “complexity”, with the highest proportions of 2-phenylethyl acetate (1.62%) and benzaldehyde (0.80%) (Table 6). The former (2-phenylethyl acetate) is associated with sweet, honey, and floral notes [59], as well as with almond and cherry notes [58], while the latter (benzaldehyde) is associated with almond and cherry notes [60]. Both may have contributed to the perceived attractiveness and aromatic “fullness” of these breads. This is consistent with the e-tongue results, showing that buckwheat variants had higher sweetness intensity (SWS) and spicy taste (SPS), and the combination of buckwheat with cistus infusion resulted in a high STS/SPS/SWS profile with low sourness (Figure 1). In summary, among the flours evaluated, the addition of hemp flour had the greatest impact on the taste profile (increased bitterness and umami), with a simultaneous increase in pyrazines potentially enhancing toasted, roasted, and earthy aroma notes. In contrast, buckwheat, despite the observed changes, maintained consumer acceptance at a level similar to that of the control bread.

However, it should be noted that the sensory evaluation conducted in this study had an exploratory character and was based on a relatively limited number of panelists, which may affect the generalizability of the results. In addition, the demographic structure of the panel (predominance of young adults and unequal gender distribution) may have influenced the observed preferences. Therefore, further studies involving larger and more diverse consumer groups are recommended to confirm the obtained findings.

## 3. Materials and Methods

### 3.1. Bread Preparation

Six wheat bread variants were produced for the purpose of this study. Three base formulations differed in flour composition: (1) WCh—wheat flour type 650 (Polskie Młyny Sp. z o.o., Warsaw, Poland) with 2.5% chia flour (Planteon Sp. z o.o., Borków Stary, Poland); (2) WChH—WCh with an additional 15% substitution of defatted hemp flour (Ol’Vita, Panków, Poland); and (3) WChB—WCh with 15% buckwheat flour (Mlevit S.A., Warsaw, Poland). All proportions were expressed relative to wheat flour mass (m/m). Corresponding variants were prepared with water fully replaced by 2.5% (*w*/*v*) *Cistus incanus* L. infusion (TAR-GROCH-FIL Sp. J., Zakliczyn, Poland) and designated WCh/Cis, WChH/Cis, and WChB/Cis.

Dough preparation followed the straight-dough method, as previously described [23,35], with modifications to accommodate the multi-ingredient formulations of the present study. Briefly, each batch comprised 975 g wheat flour, 25 g chia flour, 20 g salt (Solino S.A., Inowrocław, Poland), 30 g compressed yeast (Lesaffre Polska S.A., Wołczyn, Poland), and a variable hydration level (Table S5) determined by farinograph water absorption. Mixing was performed in an Ibis MS 130 high-speed spiral mixer (IBIS Sp. z o.o., Szubin, Poland). Following a 60 min bulk fermentation stage (21 °C, 55% RH), the dough was divided into 325 g portions and transferred to metal molds. Final proof was carried out at 32 °C and 90% RH for 30 min in a MIWE GVA fermentation chamber (MIWE Michael Wenz GmbH, Arnstein, Germany). Loaves were subsequently baked at 230 °C for 30 min in a MIWE IDEAL deck oven (MIWE Michael Wenz GmbH, Arnstein, Germany). Each formulation was produced in two independent baking replicates, each yielding 30 loaves. For analytical determinations, samples were taken from a representative composite laboratory sample obtained by combining two independent baking replicates.

### 3.2. Analysis of Basic Features

The chemical parameters of all breads were determined: ash content (AOAC Method 923.03); protein content (AOAC Method 950.36) (the protein content was calculated by applying a conversion factor of 6.25); crude fat content (AOAC Method 935.38); moisture content (AOAC Method 925.10); and total, soluble, and insoluble dietary fiber content (AOAC 991.43) [61]. Analyses were performed in triplicate.

### 3.3. Amino Acid Composition and Protein Nutritional Quality

Amino acids were determined according to the method described in [35] using ion-exchange chromatography with a strong cation ion-exchanger and a sodium-citrate elution buffer system, followed by post-column derivatization according to the standard protocol of the amino acid analyzer manufacturer (Ingos, Prague, Czech Republic). Evaluation of the acquired data was performed using Chromulan software v.0.90 (Pikron, Prague, Czech Republic).

The nutritional quality of protein was expressed as the Amino Acid Score (AAS) and calculated according to [62,63] (Equation (1)):

(1) $$AAS=\frac{{AA}_{sample}}{{AA}_{pattern}}$$

where:

*AA_sample_*—milligrams of amino acid in 1 g of test protein;

*AA_pattern_*—milligrams of amino acid in reference pattern recommended for adolescents and adults [63]. Analyses were performed in triplicate.

### 3.4. Determination of Fatty Acid Profile

The fatty acid profile was determined as fatty acid methyl esters (FAMEs), as described previously [64]. A Shimadzu GC 2010Plus gas chromatograph (Shimadzu Corp., Kyoto, Japan) with a flame ionization detector (FID) equipped with an SH-FAME column (30 m–0.32 mm–0.25 μm) was used to determine the fatty acid profile. Individual fatty acid methyl esters were identified by comparison to the standard mixture of Supelco 37 component FAME Mix, Sigma-Aldrich Co., and of conjugated linoleic acid (CLA) isomers (Sigma-Aldrich Co., St. Louis, MO, USA). Results are expressed in g/100 g of fat. Analyses were performed in duplicate.

### 3.5. Antioxidant Properties and Polyphenol Profiles

Chromatographic separation of polyphenols was performed using a NEXERA XR Shimadzu UHPLC liquid chromatograph with diode array detector (Kyoto, Japan) using an RP-18 chromatographic column (Shim-Pack Specter C18-120/3 µm, Shimadzu, Kyoto, Japan). Eluent A was a 0.1% aqueous solution of formic acid, while eluent B was a 0.1% solution of formic acid in acetonitrile. Separation was performed using gradient elution in two variants in order to achieve better separation of caffeic, vanillic and syringic acids as well as epigallocatechin gallate (Table S6). Analyses were performed in duplicate.

Total phenolic content was determined using the Folin–Ciocalteu method [65]. Samples were prepared at 0.1 g/mL concentration in water solution, as described previously [15]. Results were expressed as milligrams of gallic acid equivalent (GAE) per 100 g of product. The limits of detection and quantification were 0.3 and 0.9 mg, respectively (R^2^ = 1.0000). Measurements were performed in duplicate.

Ferric ion reducing ability was established according to work by Benzie and Strain [48]. Sample solutions (0.1 g/mL) were mixed with 1.5 mL of FRAP reagent solution and incubated at 37 °C for 30 min. Absorbance was measured (Spectro UV-VIS Dual Beam UVS-2800, Labomed, Inc., Los Angeles, CA, USA) at a wavelength of 593 nm. FRAP solution was obtained by mixing acetate buffer (pH 3.6) with 2,4,6-Tri(2-pyridyl)-s-triazine (10 mM in 40 mM HCl) and FeCl_3_ (20 mM aqueous solution) in a 10:1:1 ratio. A standard curve was made for Trolox in the range from 2 to 12 mg/L (R^2^ = 0.9981), with limits of detection and quantification of 0.2 and 0.7 mg, respectively. Results were expressed as milligrams of Trolox per 100 g of the product. Measurements were performed in duplicate.

### 3.6. Electronic Nose and Electronic Tongue Analyses

A detailed description of both methods is provided in our previous article [35]. Briefly, e-nose analysis was performed using a Heracles II electronic nose (Alpha MOS, Toulouse, France), and Kovats indices were used to identify the volatile compounds with AromaChemBase software v.8 (Alpha MOS, Toulouse, France). Each sample was measured in triplicate. Alphasoft software v.14.2 and AroChembase database (Alpha MOS, Toulouse, France) were used for instrument control, data acquisition, and data evaluation.

The Alpha MOS ASTREE II electronic tongue (e-tongue) system (Alpha MOS, Toulouse, France), was used for instrumental taste analysis of the bread samples. Sensor set #5, consisting of seven sensors (SRS, GPS, STS, SPS, UMS, SWS, and BRS), and a reference electrode (Ag/AgCl) were used. AlphaSoft software v.14.2 (Alpha MOS, Toulouse, France) was used for instrument control, data acquisition, and processing [66]. Analyses were performed in triplicate.

### 3.7. Color Analysis

Bread crumb color was measured according to the CIE L*a*b* system using a Konica Minolta CM-5 spectrophotometer (Konica Minolta Sensing, Osaka, Japan), as described previously [15]. Analyses were performed in six replicates.

### 3.8. Texture Analysis

A Texture Analyser TA.XT Plus (Stable Microsystems, Godalming, Surrey, UK) was used for the evaluation of textural parameters using the standard TPA program at a compression rate of 5 mm/s. Cylindrical bread crumb samples (10 mm diameter, 15 mm height), taken from the center of the loaf, were compressed to 50% of their original height using a P/36 probe in two cycles with a 5 s delay. The following TPA parameters were used to characterize bread crumb texture: hardness, cohesiveness, springiness, and chewiness. Calculations were performed using the Texture Exponent software v.6.0 (Stable Microsystems, Godalming, Surrey, UK). Analyses were performed in quintuplicate.

### 3.9. Consumer Acceptance

Consumer evaluation was conducted with 65 participants aged 20–30 years. Inclusion criteria included regular bread consumption (at least several times per week), no food allergies, and no taste or smell disorders. The panel comprised 51 women and 14 men. Participation was voluntary, all participants provided informed consent, and the study protocol was approved by the Independent Bioethics Committee for Research of the Medical University of Gdańsk (KB/268/2025). The study was conducted in accordance with the ethical principles of the Declaration of Helsinki (2013 revision).

Immediately prior to evaluation, the samples were assigned random three-digit codes. Sample presentation order was randomized. Evaluations were conducted in a well-lit, ventilated laboratory. Samples were presented on white plates as approximately 1 cm thick slices. Participants were provided with still mineral water to rinse their mouths between samples.

Consumer acceptance was assessed using a 7-point hedonic scale, where 1 indicated “I definitely don’t like it/I don’t like it”, and 7 indicated “I definitely like it/I like it very much”. Evaluated attributes included smell, taste, crust appearance and structure, crumb appearance and structure, and overall product quality [67].

### 3.10. Statistical Analysis

Results are presented as the mean ± standard deviation. Between-group differences were evaluated by one-way analysis of variance (ANOVA) followed by Tukey’s HSD post hoc test. Hedonic scores were analyzed using a one-way repeated-measures ANOVA (RM–ANOVA) with bread type as a within-subject factor (6 levels; N = 65). When a significant main effect was found, post hoc pairwise comparisons were performed with Holm correction for multiple testing. The significance level was set at *p* < 0.05; all statistical analyses were performed using Statistica 14.1.0.4 (TIBCO Software Inc., Palo Alto, CA, USA).

## 4. Conclusions

This study demonstrates that enriching wheat bread containing 2.5% chia flour can significantly improve its nutritional and functional value, but the final effect depends largely on the type of additive used and its associated “sensory cost”. Hemp flour significantly increased the protein and dietary fiber content of the bread, primarily through an increase in the insoluble fiber fraction. Both hemp and buckwheat flour improved the amino acid profile of bread protein, increasing lysine content and the total essential amino acids pool. Lysine remained the limiting amino acid in all variants, although its AAS value significantly increased compared to the control. Regarding the lipid profile, hemp flour most effectively increased the PUFA content and the PUFA/SFA ratio; however, the n-6/n-3 ratio also increased in hemp-enriched variants, a tradeoff that should be considered in the context of dietary recommendations for n-3 intake. Buckwheat flour shaped the lipid profile toward MUFA. Cistus infusion was a key factor in enhancing the antioxidant potential and differentiating the polyphenol profile of the bread. It increased the total content of phenolic compounds as well as TPC and FRAP values, and introduced characteristic polyphenols (e.g., EGCG, quercetin, kaempferol) into the bread matrix, which were absent in the control bread. The strongest antioxidant response was obtained in systems combining cistus infusion with hemp or buckwheat flour, with the most pronounced effect in the hemp-cistus variant, suggesting a synergistic interaction in the bread matrix. Instrumental analyses confirmed that the additives significantly altered the flavor and aroma profiles of the bread. Cistus infusion reduced sourness and enhanced umami and salty taste intensity, hemp flour increased bitterness, and buckwheat flour intensified sweet and spicy taste notes.

From a product quality perspective, the enrichment significantly affected color and texture. Hemp flour caused the greatest darkening and the highest overall color difference, while both hemp and buckwheat flours increased hardness and chewiness. Notably, the use of cistus infusion partially mitigated these textural changes, accompanied by a slightly higher crumb moisture content. The consumer acceptance results clearly showed that while hemp flour maximized nutritional benefits and improved the lipid profile parameters, it decreased hedonic acceptance, particularly for taste and overall liking. In contrast, buckwheat flour variants maintained good acceptance and were closest to the control. Incorporating cistus infusion into the hemp bread recipe improved consumer scores compared to the hemp-only variant, indicating the practical potential for better balancing nutritional functionality and sensory appeal.

In summary, hemp flour is the most effective additive when protein, fiber enrichment, and an improved PUFA lipid profile are the primary objectives. However, it requires technological strategies to reduce unfavorable sensory characteristics. Buckwheat flour offers the best compromise between improved nutritional value and consumer acceptance. Cistus infusion represents a complementary ingredient, enhancing antioxidant properties and partially mitigating undesirable textural and sensory changes, particularly in hemp-enriched bread. Further research should focus on optimizing the additive levels and baking process conditions, as well as conducting sensory evaluations in larger consumer panels, to support functional bread product development.

## Figures and Tables

**Table 1:** Chemical composition of raw material and bread samples (g/100 g of fresh matter).

	Moisture	Protein	Ash	Fat	Dietary Fiber		
					Insoluble Fraction	Soluble Fraction	Total
Raw materials							
WF	12.88 ^a^ ± 0.11	11.41 ^d^ ± 0.08	0.39 ^c^ ± 0.01	1.08 ^d^ ± 0.03	0.72 ^d^ ± 0.00	1.36 ^c^ ± 0.07	2.08 ^d^ ± 0.06
CHF	8.06 ^c^ ± 0.03	28.28 ^b^ ± 0.12	6.89 ^a^ ± 0.06	9.34 ^a^ ± 0.01	29.97 ^b^ ± 0.03	7.17 ^a^ ± 0.08	37.14 ^b^ ± 0.11
HF	7.31 ^d^ ± 0.05	33.75 ^a^ ± 0.09	6.87 ^a^ ± 0.02	7.44 ^b^ ± 0.08	39.23 ^a^ ± 0.15	1.88 ^b^ ± 0.04	41.10 ^a^ ± 0.11
BF	11.55 ^b^ ± 0.07	14.35 ^c^ ± 0.13	2.09 ^b^ ± 0.01	3.24 ^c^ ± 0.04	2.08 ^c^ ± 0.09	1.44 ^c^ ± 0.03	3.52 ^c^ ± 0.06
Bread samples							
WCh	13.96 ^a^ ± 0.30	11.39 ^c^ ± 0.01	2.48 ^e^ ± 0.01	1.42 ^c^ ± 0.01	2.01 ^b^ ± 0.03	1.46 ^b^ ± 0.07	3.47 ^c^ ± 0.08
WCh/Cis	13.42 ^a^ ± 0.12	11.41 ^c^ ± 0.02	2.75 ^d^ ± 0.01	1.39 ^c^ ± 0.01	2.03 ^b^ ± 0.07	1.44 ^b^ ± 0.08	3.47 ^c^ ± 0.01
WChH	12.57 ^b^ ± 0.11	14.86 ^a^ ± 0.11	3.39 ^a^ ± 0.00	2.27 ^a^ ± 0.02	7.72 ^a^ ± 0.01	1.96 ^a^ ± 0.06	9.67 ^a^ ± 0.06
WChH/Cis	12.73 ^ab^ ± 0.21	14.70 ^a^ ± 0.03	3.39 ^a^ ± 0.00	2.31 ^a^ ± 0.01	7.54 ^a^ ± 0.09	1.88 ^a^ ± 0.03	9.41 ^a^ ± 0.06
WChB	13.43 ^a^ ± 0.26	11.90 ^b^ ± 0.04	2.79 ^c^ ± 0.01	1.74 ^b^ ± 0.02	2.15 ^b^ ± 0.00	1.78 ^a^ ± 0.06	3.93 ^b^ ± 0.06
WChB/Cis	12.98 ^ab^ ± 0.06	11.85 ^b^ ± 0.02	2.86 ^b^ ± 0.00	1.69 ^b^ ± 0.01	2.14 ^b^ ± 0.04	1.73 ^a^ ± 0.04	3.86 ^b^ ± 0.08

Explanatory notes: WCh—wheat–chia bread (control); WCh/Cis—wheat–chia bread with cistus infusion; WChH—wheat–chia bread with hemp flour; WChH/Cis—wheat–chia–hemp bread with cistus infusion; WChB—wheat–chia bread with buckwheat flour; WChB/Cis—wheat–chia–buckwheat bread with cistus infusion; different superscript letters within a column indicate significant differences at *p* < 0.05.

**Table 2:** Amino acid profile of bread (mg/g protein).

Amino Acid	WCh	WCh/Cis	WChH	WChH/Cis	WChB	WChB/Cis
Essential amino acids (EAAs)						
Histidine	23.57 ^b^ ± 0.38	21.70 ^c^ ± 0.37	24.65 ^ab^ ± 0.21	24.70 ^a^ ± 0.37	24.00 ^ab^ ± 0.28	23.83 ^b^ ± 0.45
Isoleucine	34.48 ^b^ ± 0.10	34.34 ^b^ ± 0.34	36.21 ^a^ ± 0.35	35.81 ^a^ ± 0.22	34.82 ^b^ ± 0.07	34.98 ^b^ ± 0.50
Leucine	68.58 ^a^ ± 0.10	68.74 ^a^ ± 0.21	69.26 ^a^ ± 1.04	69.10 ^a^ ± 0.48	69.48 ^a^ ± 0.09	68.72 ^a^ ± 0.82
Lysine	23.03 ^b^ ± 0.23	23.98 ^b^ ± 1.07	27.27 ^a^ ± 0.33	27.29 ^a^ ± 0.48	28.70 ^a^ ± 0.88	28.95 ^a^ ± 1.18
Methionine	15.42 ^bc^ ± 0.00	14.80 ^c^ ± 0.44	17.09 ^a^ ± 0.32	16.98 ^a^ ± 0.47	16.12 ^ab^ ± 0.35	15.00 ^bc^ ± 0.24
Phenylalanine	46.78 ^a^ ± 0.23	46.58 ^a^ ± 0.08	47.53 ^a^ ± 0.63	47.18 ^a^ ± 0.42	47.12 ^a^ ± 0.09	47.00 ^a^ ± 0.56
Threonine	28.67 ^b^ ± 0.06	28.82 ^b^ ± 0.02	31.29 ^a^ ± 0.61	31.11 ^a^ ± 0.25	31.10 ^a^ ± 0.08	31.01 ^a^ ± 0.45
Valine	42.58 ^c^ ± 0.03	42.48 ^c^ ± 0.31	46.08 ^a^ ± 0.48	45.75 ^a^ ± 0.30	44.51 ^b^ ± 0.10	44.54 ^b^ ± 0.62
Total EAA	283.10 ^b^ ± 0.87	281.43 ^b^ ± 1.20	299.39 ^a^ ± 3.29	297.99 ^a^ ± 1.77	295.86 ^a^ ± 0.36	294.02 ^a^ ± 3.66
Non-essential amino acids (non-EAAs)						
Alanine	35.48 ^c^ ± 0.11	35.61 ^c^ ± 0.26	39.86 ^a^ ± 0.73	39.67 ^a^ ± 0.27	38.08 ^b^ ± 0.14	38.18 ^b^ ± 0.57
Arginine	43.74 ^c^ ± 0.27	45.07 ^c^ ± 0.77	68.92 ^a^ ± 1.21	68.62 ^a^ ± 0.52	54.86 ^b^ ± 0.22	55.97 ^b^ ± 0.65
Aspartic acid	49.34 ^d^ ± 0.07	50.02 ^d^ ± 0.41	70.36 ^a^ ± 1.51	70.07 ^a^ ± 0.48	55.53 ^c^ ± 0.05	60.33 ^b^ ± 0.57
Cysteine	21.99 ^ab^ ± 0.01	20.99 ^abc^ ± 0.04	19.48 ^c^ ± 0.22	20.62 ^bc^ ± 0.91	22.67 ^a^ ± 0.06	21.41 ^ab^ ± 0.75
Glutamic acid	341.83 ^a^ ± 0.15	340.91 ^a^ ± 0.57	291.73 ^c^ ± 4.36	292.82 ^c^ ± 2.34	316.79 ^b^ ± 0.26	313.89 ^b^ ± 4.04
Glycine	39.17 ^c^ ± 0.01	39.09 ^c^ ± 0.13	42.06 ^b^ ± 0.74	41.82 ^b^ ± 0.28	43.72 ^a^ ± 0.01	43.71 ^a^ ± 0.52
Proline	106.98 ^a^ ± 0.21	106.38 ^a^ ± 0.08	85.73 ^c^ ± 1.43	85.45 ^c^ ± 0.93	92.37 ^b^ ± 0.03	90.36 ^b^ ± 2.79
Serine	49.18 ^a^ ± 0.06	49.23 ^a^ ± 0.17	50.19 ^a^ ± 1.10	49.78 ^a^ ± 0.45	50.16 ^a^ ± 0.00	49.47 ^a^ ± 0.58
Tyrosine	29.20 ^c^ ± 0.04	31.28 ^ab^ ± 1.06	32.29 ^a^ ± 0.38	32.24 ^a^ ± 0.43	29.96 ^bc^ ± 0.14	32.66 ^a^ ± 0.36
Total non-EAA	716.90 ^a^ ± 0.27	718.57 ^a^ ± 2.92	700.61 ^b^ ± 11.13	701.10 ^b^ ± 4.94	704.14 ^ab^ ± 0.10	705.98 ^ab^ ± 8.20

Explanatory notes: WCh—wheat–chia bread (control); WCh/Cis—wheat–chia bread with cistus infusion; WChH—wheat–chia bread with hemp flour; WChH/Cis—wheat–chia–hemp bread with cistus infusion; WChB—wheat–chia bread with buckwheat flour; WChB/Cis—wheat–chia–buckwheat bread with cistus infusion; different superscript letters within a row indicate significant differences at *p* < 0.05.

**Table 3:** Nutritional value of protein of enriched products.

Sample	AAS [%]							
	His	Ile	Leu	Lys	Thr	Val	AAA *	SAA
WCh	147.30 ^b^ ± 2.38	114.92 ^b^ ± 0.34	112.43 ^a^ ± 0.16	47.97 ^b^ ± 0.47	114.70 ^b^ ± 0.23	106.44 ^c^ ± 0.09	185.31 ^b^ ± 0.65	162.63 ^a^ ± 0.05
WCh/Cis	135.65 ^c^ ± 2.31	114.45 ^b^ ± 1.14	112.68 ^a^ ± 0.35	49.96 ^b^ ± 2.23	115.26 ^b^ ± 0.10	106.19 ^c^ ± 0.77	189.91 ^a^ ± 2.78	155.59 ^a^ ± 1.99
WChH	154.03 ^a^ ± 1.31	120.71 ^a^ ± 1.17	113.54 ^a^ ± 1.71	56.81 ^a^ ± 0.69	125.17 ^a^ ± 2.44	115.21 ^a^ ± 1.21	194.68 ^a^ ± 2.48	158.99 ^a^ ± 2.31
WChH/Cis	154.36 ^a^ ± 2.33	119.36 ^a^ ± 0.74	113.28 ^a^ ± 0.78	56.85 ^a^ ± 0.99	124.43 ^a^ ± 1.02	114.38 ^a^ ± 0.76	193.70 ^a^ ± 2.03	159.73 ^a^ ± 3.32
WChB	150.03 ^a^ ± 1.77	116.06 ^b^ ± 0.23	113.90 ^a^ ± 0.15	59.80 ^a^ ± 1.83	124.41 ^a^ ± 0.31	111.29 ^b^ ± 0.26	187.99 ^b^ ± 0.55	164.62 ^a^ ± 1.81
WChB/Cis	148.91 ^b^ ± 2.79	116.60 ^b^ ± 1.66	112.66 ^a^ ± 1.34	60.31 ^a^ ± 2.45	124.04 ^a^ ± 1.82	111.35 ^b^ ± 1.54	194.27 ^a^ ± 2.23	158.30 ^a^ ± 4.23

Explanatory notes: WCh—wheat–chia bread (control); WCh/Cis—wheat–chia bread with cistus infusion; WChH—wheat–chia bread with hemp flour; WChH/Cis—wheat–chia–hemp bread with cistus infusion; WChB—wheat–chia bread with buckwheat flour; WChB/Cis—wheat–chia–buckwheat bread with cistus infusion; * AAA—aromatic amino acid; SAA—sulfur-containing amino acid; different superscript letters within a column indicate significant differences at *p* < 0.05.

**Table 4:** Fatty acid profiles of obtained breads (g/100 g).

Fatty Acid	WCh	WCh/Cis	WChH	WChH/Cis	WChB	WChB/Cis
C14:0	0.28 ^a^ ± 0.00	0.27 ^b^ ± 0.00	-	-	-	-
C16:0	14.95 ^a^ ± 0.01	14.65 ^a^ ± 0.10	10.35 ^d^ ± 0.00	10.36 ^d^ ± 0.02	14.39 ^b^ ± 0.21	13.69 ^c^ ± 0.01
C16:1	0.88 ^a^ ± 0.00	0.85 ^b^ ± 0.00	0.37 ^d^ ± 0.00	0.36 ^d^ ± 0.00	0.60 ^c^ ± 0.01	0.60 ^c^ ± 0.01
C18:0	2.63 ^c^ ± 0.00	2.87 ^a^ ± 0.00	2.78 ^b^ ± 0.00	2.83 ^a^ ± 0.01	2.34 ^d^ ± 0.02	2.32 ^d^ ± 0.01
C18:1 cis	13.35 ^b^ ± 0.01	12.96 ^c^ ± 0.01	11.85 ^d^ ± 0.02	11.93 ^d^ ± 0.08	22.84 ^a^ ± 0.26	22.91 ^a^ ± 0.02
C18:1 trans	0.98 ^b^ ± 0.01	0.97 ^b^ ± 0.00	0.96 ^b^ ± 0.02	0.97 ^b^ ± 0.01	1.14 ^a^ ± 0.01	1.16 ^a^ ± 0.00
C18:2 n-6 cis	44.97 ^b^ ± 0.03	44.05 ^b^ ± 0.07	51.66 ^a^ ± 0.20	51.70 ^a^ ± 0.48	41.10 ^c^ ± 0.48	41.05 ^c^ ± 0.04
C18:2 n-6 trans	0.02 ^b^ ± 0.03	0.05 ^b^ ± 0.00	0.10 ^a^ ± 0.00	0.02 ^b^ ± 0.00	0.02 ^b^ ± 0.00	-
C18:3 n-6	-	-	2.01 ^a^ ± 0.00	2.03 ^a^ ± 0.02	-	-
C18:3 n-3	21.07 ^b^ ± 0.05	22.45 ^a^ ± 0.11	18.40 ^c^ ± 0.07	18.08 ^c^ ± 0.16	14.38 ^d^ ± 0.11	14.16 ^d^ ± 0.00
C20:0	0.30 ^b^ ± 0.01	0.32 ^b^ ± 0.01	0.78 ^a^ ± 0.01	0.83 ^a^ ± 0.09	0.85 ^a^ ± 0.10	0.85 ^a^ ± 0.04
C22:6 n-3	0.57 ^c^ ± 0.06	0.56 ^c^ ± 0.05	0.56 ^c^ ± 0.01	0.33 ^d^ ± 0.07	1.56 ^b^ ± 0.06	1.72 ^a^ ± 0.01
C22:0	-	-	0.19 ^c^ ± 0.02	0.39 ^b^ ± 0.00	0.39 ^b^ ± 0.05	0.81 ^a^ ± 0.02
C24:0	-	-	-	-	0.26 ^b^ ± 0.01	0.72 ^a^ ± 0.00
SFA *	18.15 ^a^ ± 0.00	18.10 ^a^ ± 0.12	14.10 ^b^ ± 0.26	14.41 ^b^ ± 0.05	18.24 ^a^ ± 0.37	18.40 ^a^ ± 0.06
MUFA	15.21 ^b^ ± 0.00	14.78 ^b^ ± 0.01	13.18 ^c^ ± 0.00	13.27 ^c^ ± 0.10	24.58 ^a^ ± 0.28	24.67 ^a^ ± 0.03
PUFA	66.64 ^b^ ± 0.01	67.12 ^b^ ± 0.13	72.72 ^a^ ± 0.26	72.16 ^a^ ± 0.18	57.07 ^c^ ± 0.65	56.93 ^c^ ± 0.03
PUFA/SFA	3.67 ^b^ ± 0.00	3.71 ^b^ ± 0.03	5.16 ^a^ ± 0.11	5.01 ^a^ ± 0.03	3.13 ^c^ ± 0.17	3.09 ^c^ ± 0.01
n6	44.99 ^b^ ± 0.00	44.10 ^b^ ± 0.07	53.76 ^a^ ± 0.20	53.75 ^a^ ± 0.49	41.13 ^c^ ± 0.48	41.05 ^c^ ± 0.04
n3	21.64 ^a^ ± 0.01	23.02 ^a^ ± 0.06	18.96 ^b^ ± 0.06	18.41 ^b^ ± 0.31	15.94 ^c^ ± 0.17	15.88 ^c^ ± 0.01
n-6/n-3	2.08 ^c^ ± 0.00	1.92 ^c^ ± 0.00	2.84 ^a^ ± 0.00	2.92 ^a^ ± 0.08	2.58 ^b^ ± 0.00	2.59 ^b^ ± 0.00

Explanatory notes: WCh—wheat–chia bread (control); WCh/Cis—wheat–chia bread with cistus infusion; WChH—wheat–chia bread with hemp flour; WChH/Cis—wheat–chia–hemp bread with cistus infusion; WChB—wheat–chia bread with buckwheat flour; WChB/Cis—wheat–chia–buckwheat bread with cistus infusion; * SFA—saturated fatty acid; MUFA—monounsaturated fatty acid; PUFA—polyunsaturated fatty acid; different superscript letters within a row indicate significant differences at *p* < 0.05.

**Table 5:** Antioxidant compounds and antioxidant properties of bread.

Compound	WCh	WCh/Cis	WChH	WChH/Cis	WChB	WChB/Cis
Phenolic acids [mg/100 g]						
3,4-Dihydroxybenzoic acid	-	1.77 ^c^ ± 0.00	1.67 ^d^ ± 0.08	2.15 ^a^ ± 0.00	1.35 ^e^ ± 0.00	1.86 ^b^ ± 0.00
Caffeic acid	1.63 ^a^ ± 0.00	1.62 ^a^ ± 0.00	1.54 ^b^ ± 0.01	1.51 ^b^ ± 0.01	1.68 ^a^ ± 0.01	1.65 ^a^ ± 0.03
Vanillic acid	3.35 ^e^ ± 0.00	3.46 ^c^ ± 0.00	3.50 ^b^ ± 0.00	3.66 ^a^ ± 0.00	3.42 ^d^ ± 0.00	3.51 ^b^ ± 0.00
Ferulic acid	1.63 ^a^ ± 0.01	1.63 ^a^ ± 0.01	1.67 ^a^ ± 0.01	1.65 ^a^ ± 0.01	1.58 ^a^ ± 0.00	1.61 ^a^ ± 0.05
Sinapic acid	1.49 ^f^ ± 0.01	2.94 ^c^ ± 0.01	2.16 ^e^ ± 0.02	3.12 ^b^ ± 0.02	2.73 ^d^ ± 0.03	3.45 ^a^ ± 0.01
p-Coumaric acid (trans)	1.69 ^f^ ± 0.00	1.84 ^d^ ± 0.00	1.86 ^c^ ± 0.01	1.96 ^a^ ± 0.00	1.73 ^e^ ± 0.00	1.89 ^b^ ± 0.00
p-Coumaric acid (cis)	1.92 ^b^ ± 0.01	1.46 ^d^ ± 0.00	1.29 ^e^ ± 0.02	1.48 ^d^ ± 0.00	2.96 ^a^ ± 0.02	1.59 ^c^ ± 0.01
Flavan-3-ols [mg/100 g]						
Catechin	5.88 ^a^ ± 0.65	5.09 ^ab^ ± 0.12	5.33 ^ab^ ± 0.03	5.91 ^a^ ± 0.17	2.29 ^c^ ± 0.26	4.26 ^b^ ± 0.19
Epicatechin	3.13 ^d^ ± 0.10	5.85 ^b^ ± 0.01	5.44 ^b^ ± 0.05	6.22 ^b^ ± 0.02	4.20 ^c^ ± 0.03	7.75 ^a^ ± 0.48
Epigallocatechin gallate (EGCG)	-	2.01 ^a^ ± 0.01	-	1.92 ^c^ ± 0.01	1.73 ^d^ ± 0.13	1.96 ^b^ ± 0.01
Epigallocatechin	2.56 ^a^ ± 0.09	2.18 ^ab^ ± 0.15	2.39 ^ab^ ± 0.17	2.10 ^ab^ ± 0.17	1.96 ^b^ ± 0.19	2.15 ^ab^ ± 0.11
Flavonols [mg/100 g]						
Quercetin	-	2.00 ^b^ ± 0.00	1.66 ^d^ ± 0.00	1.96 ^c^ ± 0.00	1.61 ^e^ ± 0.00	2.05 ^a^ ± 0.00
Kaempferol	-	6.71 ^a^ ± 0.00	-	6.69 ^c^ ± 0.00	-	6.70 ^b^ ± 0.00
Total	23.28 ^d^ ± 0.96	38.56 ^b^ ± 0.31	28.51 ^c^ ± 0.48	40.60 ^a^ ± 0.41	27.24 ^c^ ± 0.67	40.43 ^a^ ± 0.89
Antioxidant properties						
TPC (mg GAE/L)	50.27 ^f^ ± 0.30	71.26 ^d^ ± 0.19	90.93 ^b^ ± 0.77	108.61 ^a^ ± 0.45	68.87 ^e^ ± 2.99	83.24 ^c^ ± 0.00
FRAP (mg Trolox/L)	7.82 ^f^ ± 0.17	12.66 ^c^ ± 0.05	11.49 ^d^ ± 0.01	19.71 ^a^ ± 0.11	10.32 ^e^ ± 0.01	18.67 ^b^ ± 0.10

Explanatory notes: WCh—wheat–chia bread (control); WCh/Cis—wheat–chia bread with cistus infusion; WChH—wheat–chia bread with hemp flour; WChH/Cis—wheat–chia–hemp bread with cistus infusion; WChB—wheat–chia bread with buckwheat flour; WChB/Cis—wheat–chia–buckwheat bread with cistus infusion; phenolic acids were additionally classified as hydroxybenzoic and hydroxycinnamic acid derivatives based on their chemical structures; different superscript letters within a row indicate significant differences at *p* < 0.05; TPC—total phenolic content; FRAP—ferric reducing antioxidant power; GAE—gallic acid equivalents.

**Table 6:** Major volatile aroma compounds (% of the total volatile fraction) identified in the electronic nose profile.

Compound	Content of Volatile Aroma Compounds (%)					
	WCh	WCh/Cis	WChH	WChH/Cis	WChB	WChB/Cis
Acetaldehyde	3.77 ^a^ ± 0.15	3.75 ^a^ ± 0.16	3.83 ^a^ ± 0.15	3.59 ^a^ ± 0.14	3.71 ^a^ ± 0.14	3.84 ^a^ ± 0.15
1-Propanol	1.31 ^a^ ± 0.05	1.31 ^a^ ± 0.05	1.30 ^a^ ± 0.05	1.32 ^a^ ± 0.05	1.31 ^a^ ± 0.05	1.30 ^a^ ± 0.05
2,5-Dimethylpyrazine	0.26 ^b^ ± 0.06	0.29 ^b^ ± 0.06	0.54 ^a^ ± 0.02	0.52 ^a^ ± 0.02	0.53 ^a^ ± 0.02	0.53 ^a^ ± 0.02
2-Methylpropanal	7.29 ^c^ ± 0.15	7.65 ^b^ ± 0.16	8.57 ^a^ ± 0.36	8.23 ^a^ ± 0.28	7.48 ^bc^ ± 0.18	8.41 ^a^ ± 0.37
2-Phenylethyl acetate	1.20 ^c^ ± 0.05	1.19 ^c^ ± 0.05	1.36 ^b^ ± 0.06	1.45 ^b^ ± 0.07	1.20 ^c^ ± 0.05	1.62 ^a^ ± 0.11
3-Methylbutanal	7.37 ^a^ ± 0.31	7.35 ^a^ ± 0.30	7.54 ^a^ ± 0.30	7.50 ^a^ ± 0.22	7.47 ^a^ ± 0.25	7.39 ^a^ ± 0.29
Benzaldehyde	0.44 ^c^ ± 0.02	0.44 ^c^ ± 0.02	0.63 ^b^ ± 0.02	0.44 ^c^ ± 0.02	0.49 ^c^ ± 0.02	0.80 ^a^ ± 0.06
Ethanol	72.05 ^a^ ± 0.65	71.63 ^a^ ± 0.65	69.11 ^a^ ± 0.74	70.05 ^a^ ± 0.74	71.29 ^a^ ± 0.60	68.94 ^a^ ± 0.79
Ethyl Acetate	1.32 ^b^ ± 0.05	1.31 ^b^ ± 0.05	1.39 ^a^ ± 0.06	1.41 ^a^ ± 0.06	1.32 ^b^ ± 0.05	1.31 ^b^ ± 0.05
n-butanol	1.13 ^c^ ± 0.04	1.12 ^c^ ± 0.04	1.11 ^c^ ± 0.04	1.30 ^a^ ± 0.06	1.12 ^c^ ± 0.04	1.20 ^b^ ± 0.05
Trimethylpyrazine	0.28 ^c^ ± 0.02	0.28 ^c^ ± 0.02	0.36 ^ab^ ± 0.01	0.37 ^ab^ ± 0.01	0.28 ^c^ ± 0.02	0.38 ^a^ ± 0.01

Explanatory notes: WCh—wheat–chia bread (control); WCh/Cis—wheat–chia bread with cistus infusion; WChH—wheat–chia bread with hemp flour; WChH/Cis—wheat–chia–hemp bread with cistus infusion; WChB—wheat–chia bread with buckwheat flour; WChB/Cis—wheat–chia–buckwheat bread with cistus infusion; different superscript letters within a row indicate significant differences at *p* < 0.05.

**Table 7:** Color parameters in CIE L*a*b* (D65) and color differences relative to the control (WCh).

Parameter	WCh	WCh/Cis	WChH	WChH/Cis	WChB	WChB/Cis
L*	62.10 ^a^ ± 0.87	55.75 ^b^ ± 0.28	42.11 ^d^ ± 0.59	41.37 ^d^ ± 0.54	56.92 ^b^ ± 0.40	52.30 ^c^ ± 1.49
a*	1.22 ^d^ ± 0.08	4.09 ^b^ ± 0.13	2.12 ^c^ ± 0.09	3.95 ^b^ ± 0.22	1.92 ^c^ ± 0.12	4.66 ^a^ ± 0.27
b*	14.88 ^e^ ± 0.35	20.89 ^a^ ± 0.23	16.27 ^d^ ± 0.31	18.57 ^c^ ± 0.23	16.00 ^d^ ± 0.44	20.11 ^b^ ± 0.49
ΔE	-	9.20 ^c^ ± 0.18	20.51 ^a^ ± 0.57	21.23 ^a^ ± 0.54	5.35 ^d^ ± 0.46	11.63 ^b^ ± 1.23

Explanatory notes: WCh—wheat–chia bread (control); WCh/Cis—wheat–chia bread with cistus infusion; WChH—wheat–chia bread with hemp flour; WChH/Cis—wheat–chia–hemp bread with cistus infusion; WChB—wheat–chia bread with buckwheat flour; WChB/Cis—wheat–chia–buckwheat bread with cistus infusion; different superscript letters within a row indicate significant differences at *p* < 0.05.

**Table 8:** Texture parameters and moisture of the tested bread on the day of baking.

Bread Type	Hardness [N]	Cohesiveness [-]	Springiness [-]	Chewiness [N]	Moisture [%]
WCh	15.47 ^c^ ± 0.35	0.85 ^a^ ± 0.02	1.00 ^a^ ± 0.00	13.06 ^b^ ± 0.70	41.42 ^b^ ± 0.09
WCh/Cis	13.19 ^d^ ± 0.04	0.85 ^a^ ± 0.01	1.00 ^a^ ± 0.00	11.13 ^c^ ± 0.60	42.58 ^ab^ ± 0.17
WChH	19.85 ^ab^ ± 0.77	0.81 ^b^ ± 0.00	0.95 ^b^ ± 0.00	16.97 ^a^ ± 0.99	42.92 ^ab^ ± 0.48
WChH/Cis	18.96 ^b^ ± 0.09	0.83 ^ab^ ± 0.02	1.00 ^a^ ± 0.00	15.57 ^ab^ ± 0.22	43.22 ^ab^ ± 0.82
WChB	22.05 ^a^ ± 0.78	0.82 ^ab^ ± 0.01	1.00 ^a^ ± 0.00	16.92 ^a^ ± 0.66	43.18 ^ab^ ± 0.71
WChB/Cis	16.87 ^c^ ± 1.30	0.86 ^a^ ± 0.02	1.00 ^a^ ± 0.00	13.33 ^b^ ± 0.59	43.90 ^a^ ± 0.25

Explanatory notes: WCh—wheat–chia bread (control); WCh/Cis—wheat–chia bread with cistus infusion; WChH—wheat–chia bread with hemp flour; WChH/Cis—wheat–chia–hemp bread with cistus infusion; WChB—wheat–chia bread with buckwheat flour; WChB/Cis—wheat–chia–buckwheat bread with cistus infusion; different superscript letters within a column indicate significant differences at *p* < 0.05.

**Table 9:** Acceptance assessment of bread.

Sample	Smell	Taste	Appearance and Structure of the Crust	Appearance and Structure of the Crumb	Overall Acceptance
WCh	4.81 ^a^ ± 1.22	4.75 ^a^ ± 1.10	4.85 ^a^ ± 1.22	4.57 ^a^ ± 1.28	4.74 ^a^ ± 1.08
WCh/Cis	4.72 ^a^ ± 1.17	4.58 ^ab^ ± 1.14	4.72 ^a^ ± 1.08	4.60 ^a^ ± 1.10	4.75 ^a^ ± 1.06
WChH	3.45 ^b^ ± 1.53	2.80 ^d^ ± 1.39	3.82 ^b^ ± 1.13	3.20 ^c^ ± 1.30	3.25 ^b^ ± 1.17
WChH/Cis	3.89 ^b^ ± 1.17	3.35 ^c^ ± 1.50	4.05 ^b^ ± 1.29	4.02 ^b^ ± 1.35	3.49 ^b^ ± 1.08
WChB	4.52 ^a^ ± 1.42	4.55 ^ab^ ± 1.17	4.68 ^a^ ± 1.35	4.26 ^ab^ ± 1.05	4.46 ^ab^ ± 1.09
WChB/Cis	4.68 ^a^ ± 1.29	4.25 ^b^ ± 1.36	4.45 ^a^ ± 1.15	4.20 ^ab^ ± 1.33	4.32 ^b^ ± 1.15

Explanatory notes: WCh—wheat–chia bread (control); WCh/Cis—wheat–chia bread with cistus infusion; WChH—wheat–chia bread with hemp flour; WChH/Cis—wheat–chia–hemp bread with cistus infusion; WChB—wheat–chia bread with buckwheat flour; WChB/Cis—wheat–chia–buckwheat bread with cistus infusion; different superscript letters within a column indicate significant differences at *p* < 0.05.

## Data Availability

The original contributions presented in this study are included in the article and Supplementary Materials. Further inquiries can be directed to the corresponding author.

## Supplementary Materials

The following supporting information can be downloaded at: https://www.mdpi.com/article/10.3390/molecules31071198/s1, Table S1: Amino acid profile of raw materials (mg/g protein); Table S2: Fatty acid profile of raw materials; Table S3: Antioxidant compounds in *Cistus incanus* sample (mg/100 g); Table S4: Antioxidant compounds in flours (mg/100 g); Table S5: Raw material composition of bread; Table S6: Gradient separation parameters; Table S7. Validation parameters for polyphenol analysis.

## Abbreviations

The following abbreviations are used in this manuscript:

WF	Wheat flour
ChF	Chia flour
HF	Hemp flour
BF	Buckwheat flour
WCh	Wheat–chia bread (control)
WCh/Cis	Wheat–chia bread with water replaced by cistus infusion
WChH	Wheat–chia bread with the addition of hemp flour
WChH/Cis	Wheat–chia–hemp bread with cistus infusion instead of water
WChB	Wheat–chia bread with the addition of buckwheat flour
WChB/Cis	Wheat–chia–buckwheat bread with cistus infusion instead of water
