# Supplementary material for: Enrichment of Wheat–Chia Bread with Hemp, and Buckwheat Flours and Cistus incanus L. Infusion: Impact on Chemical Composition, Polyphenols, Fatty Acids, Amino Acids, and Consumer Acceptance
Source: Molecules. 2026 Apr 3;31(7):1198. doi: 10.3390/molecules31071198 (PMC13074323; doi:10.3390/molecules31071198)
Supplement: Supplementary file 1 [file molecules-31-01198-s001.zip › molecules-4199171-supplementary.pdf]

molecules

MDPI

Table S1. Amino acid profile raw material (mg/g protein).

Amino acid	WF	ChF	HF	BF
Essential amino acids (EAA)				
Histidine	23.04^{c} ± 0.79	28.90^{ab} ± 1.16	29.75^{a} ± 1.54	26.23^{b} ± 0.19
Isoleucine	32.75^{b} ± 0.60	35.34^{b} ± 1.43	40.70^{a} ± 2.29	35.65^{b} ± 0.17
Leucine	67.15^{a} ± 1.26	66.39^{a} ± 2.77	69.35^{a} ± 3.77	66.44^{a} ± 0.49
Lysine	20.79^{d} ± 0.47	50.42^{b} ± 2.09	37.32^{c} ± 2.03	62.37^{a} ± 0.31
Methionine	14.66^{c} ± 0.50	38.25^{a} ± 3.70	24.47^{b} ± 0.22	17.95^{c} ± 0.42
Phenylalanine	47.89^{a} ± 1.14	50.65^{a} ± 2.18	47.08^{a} ± 2.73	45.74^{a} ± 0.18
Threonine	25.10^{c} ± 0.47	36.80^{ab} ± 1.47	35.48^{b} ± 1.78	39.54^{a} ± 0.23
Valine	39.48^{b} ± 1.00	48.31^{a} ± 2.22	52.81^{a} ± 3.17	50.91^{a} ± 0.40
Total EAA	270.85^{b} ± 6.09	355.05^{a} ± 9.89	336.97^{a} ± 17.13	344.82^{a} ± 2.40
Endogenous amino acids (non-EAA)				
Alanine	29.01^{c} ± 0.57	51.29^{a} ± 2.19	46.84^{b} ± 2.44	46.80^{ab} ± 0.27
Arginine	36.88^{c} ± 0.62	105.93^{b} ± 3.99	126.99^{a} ± 7.70	107.79^{b} ± 0.65
Aspartic acid	42.11^{d} ± 0.71	87.33^{c} ± 3.38	112.33^{a} ± 5.73	101.44^{b} ± 0.65
Cysteine	20.96^{b} ± 0.55	26.52^{a} ± 1.88	16.71^{c} ± 0.11	22.21^{b} ± 0.54
Glutamic acid	371.65^{a} ± 7.18	193.67^{b} ± 8.30	189.31^{b} ± 10.92	197.52^{b} ± 1.15
Glycine	34.76^{c} ± 0.63	50.95^{b} ± 2.14	47.42^{b} ± 2.37	63.73^{a} ± 0.52
Proline	119.28^{a} ± 3.22	34.33^{b} ± 2.05	34.95^{b} ± 0.28	33.65^{b} ± 0.81
Serine	47.39^{b} ± 1.09	56.51^{a} ± 2.16	51.26^{b} ± 2.72	52.45^{ab} ± 0.47
Tyrosine	27.11^{b} ± 0.47	38.41^{a} ± 1.38	37.22^{a} ± 2.74	29.59^{b} ± 0.00
Total non-EAA	729.15^{a} ± 14.07	644.95^{b} ± 23.71	663.03^{b} ± 34.99	655.18^{b} ± 5.08

Explanatory notes:

Explanatory notes:

WF – wheat flour; ChF – chia flour; HF – hemp flour; BF – buckwheat flour; different superscript letters within a row indicate significant differences at $p < 0.05$

Table S2. Fatty acids profile of raw materials.

Fatty acid	WF	ChF	HF	BF
C16:0	17.86^{a} ± 0.06	7.35^{c} ± 0.06	7.07^{d} ± 0.03	14.07^{b} ± 0.10
C18:0	1.03^{d} ± 0.01	2.74^{b} ± 0.01	2.96^{a} ± 0.04	2.04^{c} ± 0.03
C18:1 cis	10.71^{c} ± 0.00	5.93^{d} ± 0.01	11.72^{b} ± 0.06	35.78^{a} ± 0.10
C18:1 trans	0.91^{b} ± 0.00	0.77^{c} ± 0.02	0.90^{b} ± 0.00	1.30^{a} ± 0.01
C18:2 n-6 cis	64.24^{a} ± 0.05	20.18^{d} ± 0.14	56.05^{b} ± 0.01	35.86^{c} ± 0.06
C18:3 n-6	–	–	3.29^{a} ± 0.01	–
C18:3 n-3	4.18^{c} ± 0.02	62.49^{a} ± 0.05	15.84^{b} ± 0.02	1.92^{d} ± 0.01
C20:0	–	0.41^{c} ± 0.00	1.17^{b} ± 0.01	1.89^{a} ± 0.01
C22:6 DHA n-3	0.58^{b} ± 0.01	–	0.51^{c} ± 0.01	3.63^{a} ± 0.18
C22:0	0.24^{c} ± 0.00	–	0.49^{b} ± 0.00	2.13^{a} ± 0.01
C24:0	0.26^{b} ± 0.00	–	–	1.37^{a} ± 0.02
SFA*	19.38^{b} ± 0.07	10.51^{d} ± 0.06	11.69^{c} ± 0.00	21.51^{a} ± 0.04
MUFA	11.62^{c} ± 0.01	6.71^{d} ± 0.03	12.62^{b} ± 0.06	37.08^{a} ± 0.09
PUFA	69.01^{c} ± 0.07	82.67^{a} ± 0.09	75.69^{b} ± 0.06	41.41^{d} ± 0.13
PUFA/SFA	3.56^{c} ± 0.02	7.87^{a} ± 0.03	6.48^{b} ± 0.01	1.93^{d} ± 0.01
n6	64.24^{a} ± 0.05	20.18^{d} ± 0.14	59.34^{b} ± 0.02	35.86^{c} ± 0.06
n3	4.77^{d} ± 0.02	62.49^{a} ± 0.05	16.35^{b} ± 0.03	5.55^{c} ± 0.19
n-6/n-3	13.48^{a} ± 0.06	0.32^{d} ± 0.00	3.63^{c} ± 0.01	6.46^{b} ± 0.23

Explanatory notes:

Explanatory notes:
WF – wheat flour; ChF – chia flour; HF – hemp flour; BF – buckwheat flour ; *SFA–saturated fatty acids; MUFA–monounsaturated fatty acids; PUFA–polyunsaturated fatty acids; different superscript letters within a raw indicate significant differences at $p < 0.05$

Table S3. Antioxidant compounds in *Cistus incanus* sample (mg/100 g).

**Compound (mg /100 g)**	**Cistus**
Phenolic acids	
Gallic acid	2447.01 ± 1.05
3,4-Dihydroxybenzoic acid	131.78 ± 3.10
Chlorogenic acid	8.73 ± 0.81
Caffeic acid	9.07 ± 0.87
Vanillic acid	112.50 ± 7.85
Syringic acid	–
Ferulic acid	27.50 ± 1.31
Sinapic acid	19.17 ± 0.85
Ellagic acid	–
p-Coumaric acid (trans)	24.93 ± 0.27
p-Coumaric acid (cis)	90.03 ± 0.16
Flavan-3-ols	
Catechin	12.81 ± 3.13
Epicatechin	21.39 ± 5.33
Epigallocatechin gallate (EGCG)	4.32 ± 0.01
Epigallocatechin	183.14 ± 30.59
Flavonols	
Quercetin	10.09 ± 0.01
Kaempferol	8.13 ± 0.00
Total	3111.60 ± 32.41
FRAP* (mg Trolox/ L r. 2.5%)	580.61 ± 0.52
TPC (mg GAE/ L r. 2.5.%)	1810.67 ± 50.10

Explanatory notes:
Phenolic acids were additionally classified as hydroxybenzoic and hydroxycinnamic acid derivatives based on their chemical structures; FRAP – ferric reducing antioxidant power; TPC – total phenolic content; GAE – gallic acid equivalents

Table S4 .Antioxidant compounds in flours (mg/100 g).

**Compound (mg/100 g)**	**WF**	**ChF**	**HF**	**BF**
Phenolic acids				
3,4-Dihydroxybenzoic acid	–	$1.99^{c} \pm 0.00$	$2.77^{b} \pm 0.97$	$3.73^{a} \pm 0.02$
Caffeic acid	–	$1.81^{b} \pm 0.00$	$1.79^{b} \pm 0.07$	$2.61^{a} \pm 0.00$
Vanillic acid	$3.26^{d} \pm 0.00$	$3.82^{c} \pm 0.00$	$3.92^{b} \pm 0.01$	$4.02^{a} \pm 0.03$
Ferulic acid	$1.37^{c} \pm 0.00$	$1.74^{b} \pm 0.00$	$2.09^{a} \pm 0.01$	$2.07^{a} \pm 0.03$
Sinapic acid	$1.06^{c} \pm 0.01$	$160.05^{a} \pm 0.57$	$2.09^{b} \pm 0.02$	$1.07^{c} \pm 0.02$
p-Coumaric acid (trans)	$1.70^{c} \pm 0.01$	$1.73^{c} \pm 0.02$	$2.35^{a} \pm 0.03$	$1.87^{b} \pm 0.00$

https://doi.org/10.3390/molecules31071198

p-Coumaric acid (cis)	1.55^{c} ± 0.01	1.51^{c} ± 0.01	1.99^{b} ± 0.00	2.36^{a} ± 0.04
Flavan-3-ols				
Catechin	–	2.92^{b} ± 0.04	0.25^{c} ± 0.02	8.59^{a} ± 0.45
Epicatechin	0.02^{d} ± 0.00	2.31^{c} ± 0.02	8.10^{b} ± 0.03	12.69^{a} ± 1.11
Epigallocatechin gallate	–	2.11^{a} ± 0.08	1.83^{b} ± 0.03	2.07^{a} ± 0.07
Epigallocatechin	–	–	–	20.49^{a} ± 2.64
Flavonols				
Quercetin	–	1.58^{b} ± 0.00	1.99^{a} ± 0.00	1.56^{c} ± 0.00
Kaempferol	–	6.61^{b} ± 0.00	6.67^{a} ± 0.00	–
Total	8.96^{d} ± 0.02	188.18^{a} ± 0.58	35.93^{c} ± 0.98	63.13^{b} ± 2.90
FRAP (mg trolox/ kg^{-1})	1.67^{c} ± 0.04	101.91^{a} ± 0.15	33.01^{b} ± 1.31	33.44^{b} ± 0.09
TPC (mg GAE/kg^{-1})	47.97^{d} ±0.48	344.16^{a} ± 2.24	285.54^{b} ± 1.17	215.23^{c} ± 0.94

Explanatory notes:

Explanatory notes:

WF – wheat flour; ChF – chia flour; HF – hemp flour; BF – buckwheat flour; phenolic acids were additionally classified as hydroxybenzoic and hydroxycinnamic acid derivatives based on their chemical structures; different superscript letters within a row indicate significant differences at $p < 0.05$; FRAP – ferric reducing antioxidant power; TPC – total phenolic content; GAE – gallic acid equivalents

Table S5. Raw material composition of bread.

	Unit	WCh	WCh/Cis	WChH	WChH/Cis	WChB	WChB/Cis
% replacement of WF				15	15	15	15
WF	[g]	975	975	825	825	825	825
ChF		25	25	25	25	25	25
HF		-	-	150	150	-	-
BF		-	-	-	-	150	150
Yeast		30	30	30	30	30	30
Salt		20	20	20	20	20	20
Water	[mL]	581	-	603	-	587	-
Cistus infusion		-	581	-	603	-	587

Explanatory notes:

WF – wheat flour; ChF – chia flour; HF – hemp flour; BF – buckwheat flour. Bread variants are defined in the Abbreviations section. Hemp flour (HF) and buckwheat flour (BF) were used as partial replacements of wheat flour at a level of 15% (w/w of total flour basis). Chia flour (ChF) was added at a constant level across all formulations.

Table S6. Gradient separation parameters.

Time [min]	Eluent A [%]
Method 1	
0	17
3	17
3.5	20
12	20
12.5	40
20	40
20.5	90
26.5	90
30	17
Method 2	
0	10
9	10
10	15
15	15
16	30
40	30
41	90
53	90
58	10

Table S7. Validation parameters for polyphenol analysis

Name	Wave length [nm]	LOD [µg/mL]	LOQ [µg/mL]	Parameters of calibration curve		R^2	concentration range [µg/g]
				a	b		
Gallic acid	270	5.17	15.67	10246.8	-20215.7	0.9988	16.5-124
3,4-Dihydroxybenzoic acid	260	2.32	7.04	41541.8	-32001	0.9999	21-210
Chlorogenic acid	320	2.25	6.83	22248.5	569289.5	0.9998	15-155
Caffeic acid	320	0.39	1.17	66314.7	-215347	0.9999	24.5-245
Vanillic acid	260	0.80	2.42	45186.5	-243482	0.9999	21-157.5
Ferulic acid	320	0.22	0.66	59093.8	-84609.4	0.9999	14-145
Sinapic acid	320	0.05	0.16	46873.1	-82248.7	1.0000	18-90
p-Coumaric acid (trans)	300	0.40	1.21	83195. 4	-135441	0.9999	21-210
p-Coumaric acid (cis)	300	0.13	0.38	44844.7	-77901.9	1.0000	21.5-161
Catechin	275	0.96	2.89	4935.074	4492.5	0.9999	18-139
Epicatechin	275	1.56	4.74	5920.091	-5531.11	0.9999	16-160
Epigallocatechin gallate (EGCG)	275	0.32	0.97	12880.1	-101825	0.9999	20-150
Epigallocatechin	270	0.50	1.50	1766.8	4167.5	0.9999	17.5-175
Quercetin	370	0.94	2.86	44707.9	-48471.5	0.9999	16-160
Kaempferol	370	0.05	0.15	45803.8	-98714.7	1.0000	13-100

https://doi.org/10.3390/molecules31071198
